# Inflammatory therapeutic targets in coronary atherosclerosis—from molecular biology to clinical application

**DOI:** 10.3389/fphys.2014.00455

**Published:** 2014-11-21

**Authors:** Fabian Linden, Gabriele Domschke, Christian Erbel, Mohammadreza Akhavanpoor, Hugo A. Katus, Christian A. Gleissner

**Affiliations:** Department of Cardiology, University of HeidelbergHeidelberg, Germany

**Keywords:** atherosclerosis, inflammation, clinical trials, coronary artery disease, prevention

## Abstract

Atherosclerosis is the leading cause of death worldwide. Over the past two decades, it has been clearly recognized that atherosclerosis is an inflammatory disease of the arterial wall. Accumulating data from animal experiments have supported this hypothesis, however, clinical applications making use of this knowledge remain scarce. In spite of optimal interventional and medical therapy, the risk for recurrent myocardial infarction remains by about 20% over 3 years after acute coronary syndromes, novel therapies to prevent atherogenesis or treat atherosclerosis are urgently needed. This review summarizes selected potential molecular inflammatory targets that may be of clinical relevance. We also review recent and ongoing clinical trails that target inflammatory processes aiming at preventing adverse cardiovascular events. Overall, it seems surprising that translation of basic science into clinical practice has not been a great success. In conclusion, we propose to focus on specific efforts that promote translational science in order to improve outcome and prognosis of patients suffering from atherosclerosis.

## Atherosclerosis

Atherosclerosis and its consequences remain a major global challenge. Coronary artery or cerebrovascular disease represent the leading cause of mortality worldwide (Roger et al., 2011; Go et al., 2014). Apart from its lethal consequences such myocardial infarction or stroke, atherosclerosis underlies the cause of a huge burden of morbidity, associated with heart failure due to ischemic heart disease or neurological impairment due to stroke.

Over the past decades, therapeutic options to treat atherosclerosis or at least prevent its consequences have been significantly improved including new pharmacological treatments such as novel inhibitors of platelet aggregation (Cannon et al., 2010) but also interventional techniques such as percutaneous coronary interventions (Meier and Timmis, 2012). However, there is still an enormous unmet need: after 3 years, 20% of ACS patients suffer from recurrent myocardial infarction despite optimal medial therapy (Cannon et al., 2005; Stone et al., 2011). Thus, novel treatment options are desperately needed in order to reduce morbidity and mortality due to atherosclerosis (Libby et al., 2011).

## A short historical review—pathophysiological concepts of atherosclerosis and currently unmeet needs

We currently believe that atherosclerosis is an inflammatory disease (Lusis, 2000; Glass and Witztum, 2001; Woollard and Geissmann, 2010; Zernecke and Weber, 2010; Libby et al., 2011). There is clear evidence that modern lifestyle with high fat diet and lack of physical activity may promote atherosclerotic disease, however, atherosclerosis has been around for millennia (Leibowitz, 1970; Murphy et al., 2003). In fact, atherosclerosis and its clinical consequences have been a longstanding problem of mankind.

Serious research investigating the underlying causes began in the 18th century. In 1793, Edward Jenner described hardening and calcification of vessels (Leibowitz, 1970). In 1913, Nikolai Anitschkow found that lipid deposition plays an important role in vascular disease (Syverson von Gemminge-Guttenberg, 2013). Over the past decades, epidemiological studies such as the Framingham Study have helped us to identify important cardiovascular risk factors including arterial hypertension or diabetes mellitus (Hense, 2003).

It is surprising that even though Rudolph Virchow had suggested atherosclerosis to be an inflammatory condition as early as in 1856 (Virchow, 1856). It took more than a century until this hypothesis was accepted and lead to advances of atherosclerosis research. The idea that inflammatory processes may substantially contribute to the disease process was largely forgotten. In the mid-seventies, atherosclerosis was still thought to be caused by mechanical or chemical endothelial injury leading to loss of endothelial integrity, adherence of platelets to the subendothelial matrix accompanied by release of humoral factors inducing proliferation of smooth muscle cells (Ross, 1976). In the mid-eighties, the important role of the immune system and inflammatory processes during atherogenesis and disease progression was recognized (Hansson, 1993).

Nevertheless, atherosclerosis remains an epidemiologically highly prevalent disease and myocardial infarction and stroke represent the most important causes of death worldwide (Roger et al., 2011; Go et al., 2014). Furthermore, despite great advances in treating acute coronary syndromes and controlling risk factors leading to coronary artery disease, there is still an enormous need for further therapies as even with optimal medical treatment (including platelet inhibitors, beta blockers, ACE inhibitors and statins) the recurrent rate of acute coronary syndromes remains at 20% within 3 years (Cannon et al., 2005; Stone et al., 2011). This creates a great burden of disease, reduction of life quality, and a great financial pressure on societies, which need to fund treatment and consequences of myocardial infarction and stroke (Sampson et al., 2012; Everett et al., 2013; Sirimarco et al., 2014). In summary, the need for novel targeted treatment options that may help to prevent cardiovascular events is evident.

## Rationale for anti-inflammatory therapy of atherosclerosis

There are several reasons that make anti-inflammatory therapy a reasonable and promising approach to treat atherosclerotic disease:

1. There is a large body of experimental evidence that atherosclerosis is an inflammatory disease (Lusis, 2000; Glass and Witztum, 2001; Woollard and Geissmann, 2010; Zernecke and Weber, 2010; Libby et al., 2011).
2. The role of inflammation is underscored by the relevance of inflammatory markers in assessing the prognosis of the disease. As reviewed by Ridker, when looking at the most important biomarkers predicting outcome in cardiovascular disease, markers indicating inflammation clearly prevail (Blake and Ridker, 2001). Among these are interleukin-6, soluble ICAM-1, serum amyloid-A, and C-reactive protein.
3. Anti-inflammatory therapies (e.g., to treat rheumatoid arthritis) have been associated with reduction of atherosclerotic disease burden (Ridker, 2009).

## Basic mechanisms of inflammation in atherosclerosis

To decide which elements of the inflammatory cascade may represent optimal targets, one has to be aware of the basic components of the inflammatory process and their role during the disease process. These components have been excellently reviewed recently (Tabas and Glass, 2013).

Briefly, the inflammatory reaction comprises four functional levels:

1. *Inducers* including pattern-associated molecular patterns (PAMPs) or danger-associated molecular patterns (DAMPs): One has to differentiate between mechanical factors such as shear stress (Jongstra-Bilen et al., 2006) and biochemical factors such as low density lipoproteins (LDL) or their components (i.e., triglycerides, cholesterol esters, phospholipids, free cholesterol, and apolioproteins) (Gleissner et al., 2007). Furthermore, there are factors that may involve biochemical processes leading to physical damage such as oxidative stress (Victor et al., 2009).
2. *Receptors* such as Toll-like receptors or scavenger receptors: A large number of receptors involved in atherogenesis has been discussed, most prominently scavenger receptors such as CD36 or SR-A mediating the uptake of modified LDL by macrophages, which in turn become foam cells (Shashkin et al., 2005).
3. The level of *signal transduction* involving various types of kinases, but also transcription factors such as NF-kB;
4. *Effectors* of inflammation leading to resolution of inflammation, tissue damage, or the immune response: In this context, a large number of cytokines has been proposed to play an important role for atherogenesis including IL-6, TNF-α, IL-1β, or IL-17 (Erbel et al., 2009, 2010; Micha et al., 2011; IL6R Genetics Consortium Emerging Risk Factors Collaboration et al., 2012).

These four levels are orchestrated by a plethora of amplifiers such as cytokines and chemokines that modulate each step and generate a delicate equilibrium.

In animal models, numerous pro- or anti-inflammatory molecules have been demonstrated to be important during atherogenesis based on data from knock out, knock in or bone marrow transplant models. However, the number of therapeutic targets that have actually made it into clinical practice is disappointingly low. This may be due to a number of reasons: (1) Animal models generally study lesion development. By contrast, in clinical practice patients present with established atherosclerosis. Thus, the therapeutic aim is lesion stabilization (or regression) rather than preventing lesion development (Libby and Aikawa, 2002). (2) Many animal models do not properly reflect human disease—e.g., mice do not display plaque rupture, a key feature of human unstable atherosclerosis. (3) Finally, the discrepancies may result from the fact that there are substantial differences between murine and human inflammation (Seok et al., 2013).

So, while we believe to have understood the basic principles of atherogenesis, there is still a large gap between basic science results derived from animal models and feasible anti-inflammatory therapies in the clinical setting.

## Current studies testing anti-inflammatory agents to treat atherosclerosis

The following paragraphs provide an overview of recently completed or ongoing investigations into the effects of modulators of inflammation on cardiovascular disease.

There are three major challenges when studying anti-atherosclerotic therapies in clinical trials: (1) Mechanisms that have proven to promote atherogenesis in animal models may not be suitable to prevent lesion progression or destabilization in humans. One example is Acyl-CoA:cholesterol acyltransferase (ACAT) inhibition, which successfully reduces atherosclerosis in mice or rabbits, while failing to do so in humans (Kitayama et al., 2006; Terasaka et al., 2007; Tardif et al., 2008). (2) In clinically stable patients, the event rate is usually quite low unless patient numbers are high and observation times are long. This translates into a lengthy clinical trial period and high cost for investigating anti-atherosclerosis drugs. (3) To circumvent this problem, many studies have used surrogate parameters such as intima media thickness, plasma levels of specific markers (e.g., HDL or CRP) or plaque volume etc. as primary end points. However, in many cases these surrogate markers have been found not to properly reflect cardiovascular outcome as seen with substantially increased HDL levels without decrease in adverse cardiovascular events during treatment with dalcetrapib (Schwartz et al., 2012), a cholesterylester transfer protein (CETP) inhibitor.

We therefore specifically focus on those studies that investigate cardiovascular outcome. Applying these criteria substantially reduces the number of relevant trials (Table 1), however, it seems to be the only way to get trustworthy results.

**Table 1:** **Summary of selected trials investigating anti-inflammatory agents in regards to prevention of adverse cardiovascular events in patients with established coronary artery disease**.

**Trial name, acronym**	**Basic principle**	**Intervention**	**Patient number**	**Inclusion criteria**	**Primary end points**	**Duration**	**Outcome, references**
**MODULATION OF INDUCERS**							
Aggressive Reduction of Inflammation Stops Events	Antioxidative threrapy	Succinobucol vs. placebo	6000	ACS (14 days to 12 months prior to randomization) and diabetes or age >55 years plus low HDL/previous MI/diagnosed CAD/prior congestive heart failure/LVEF <40%	All-cause death	June 2003 through December 2006	Succinbucol had no effect on the primary endpoint (Tardif et al., 2008)
ARISE							
Investigation of Lipid Level Management to Understand Its Impact in Atherosclerotic Events	CETP inhibition	Torcetrapib + atorvastatin vs. atorvastatin	15,067	History of CAD (MI, stroke, ACS, peripheral vascular disease, and cardiac revascularization) 30 days to 5 years before screening	Death from CAD, nonfatal MI, stroke, or hospitalization for unstable angina	March 2008 through November 2012	Torcetrapib increased HDL levels, decreased LDL levels, increased blood pressure, increased cardiovascular mortality (Kuhnast et al., 2014)
ILLUMINATE							
A Study of RO4607381 in Stable Coronary Heart Disease Patients With Recent Acute Coronary Syndrome	CETP inhibition	Optimal medical therapy—dalcetrapib vs. optimal medical therapy + placebo	15,871	Recent ACS	Death from CAD, nonfatal MI, ischemic stroke, unstable angina, or cardiac arrest with resuscitation	April 2008 through July 2010	Dalcetrapib increased HDL levels but did not reduce cardiovascular events (Kitayama et al., 2006)
Dal-OUTCOMES							
Randomized EValuation of the Effects of Anacetrapib Through Lipid-modification	CETP inhibition	Anacetrapib vs. placebno	30,000	Age >50 years, history of MI or cerebrovascular disease or peripheral vascular disease or diabetes with symptomatic heart disease	Coronary death, MI or coronary revascularization procedure	June 2011 through June 2017	Not published yet (Toth et al., 2013)
REVEAL							
STabilization Of Atherosclerotic Plaque By Initiation of DarapLadIb TherapY	Inhibition of lipoprotein-associated phospholipase A2 (Lp-PLA_2_)	Optimal medical therapy + Darapladib vs. optimal medical therapy + placebo	15,828	Stable CAD plus one risk factor (age >60 years, diabetes mellitus, low HDL, tobacco, renal failure)	Cardiovascular death, non-fatal MI, stroke	December 2008 through October 2013	Darpladib did not reduce the composite endpoint (White et al., 2010)
STABILITY							
Stabilization Of pLaques usIng Darapladib— Thrombolysis in Myocardial Infarction 52	Inhibition of lipoprotein-associated phospholipase A2 (Lp-PLA_2_)	Optimal medical therapy + Darapladib vs. optimal medical therapy + placebo	13,000	ACS within 30 days prior to inclusion plus one risk factor (prior MI, age >60 years, diabetes mellitus, renal failure, peripheral vascular disease, stroke)	Cardiovascular death, non-fatal MI, stroke	December 2009 through March 2014	No results published yet
SOLID-TIMI52							
Efficacy of Pioglitazone on Macrovascular Outcome in Patients With Type 2 Diabetes	Peroxisome proliferator-activated receptor agonist	Pioglitazone vs. Placebo	4373	Diabetes mellitus plus prior MI, percutaneous coronary intervention or coronary artery bypass graft or stroke (within 6 months); prior ACS (within 3 months); peripheral arterial obstructive disease; objective ecidence of coronary artery disease	Time to all cause mortality, non-fatal MI, stroke, ACS, major leg amputation, cardiac Intervention, bypass surgery or leg revascularization	May 2001 through January 2005	Pioglitazone reduces the composite of all-cause mortality, non-fatal myocardial infarction, and stroke in patients with type 2 diabetes who have a high risk of macrovascular events (Nissen and Wolski, 2007)
PROactive							
Rosiglitazone evaluated for cardiovascular outcomes in oral agent combination therapy for type 2 diabetes (RECORD): a multicentre, randomized, open-label trial	Peroxisome proliferator-activated receptor-γ agonist	Rosiglitazone vs. Placebo	4447	Diabetes mellitus plus	Combined endpoint of cardiovascular death and/or cardiovascular hospitalization	April 2001 through December 2008	Although the data are inconclusive about any possible effect on myocardial infarction, rosiglitazone does not increase the risk of overall cardiovascular morbidity or mortality compared with standard glucose-lowering drugs (Neve et al., 2000)
Treatment of HDL to Reduce the Incidence of Vascular Events	Niacin	Niacin/laropiprant with simvastatin or ezitimibe/simvastatin vs. placebo with simvastatin or ezitimibe/simvastatin	25,673	History of MU, cerebrovascular disease, peripheral artery disease, diabetes mellitus with any of the above or with evidence for CAD	Time to first major vascular event (non-fatal MI, coronary death, non-fatal stroke or revascularization)	January 2007 through October 2012	The addition of extended-release niacin–laropiprant to statin-based LDL cholesterol–lowering therapy did not significantly reduce the risk of major vascular events but did increase the risk of serious adverse events
HPS2-THRIVE							
**MODULATION OF INDUCERS**							
Canakinumab Anti-inflammatory Thrombosis Outcome Study	Anti-IL1β antibody	Caniakinumab vs. Placebo	17,200	Prior MI (>30 days), hs-CRP >2mg/l	Cardiovascular death, non-fatal MI, stroke	April 2011 through July 2016	Not published yet (Ridker et al., 2012)
CANTOS							
Cardiovascular Inflammation Reduction Trial	Low dose methotrexate	Methotrexate (+folate) vs. placebo (+folate)	7000	Prior MI (within 5 years), at least 60 days stable, diabetes/metabolic syndrome	Cardiovascular death, non-fatal MI, stroke	April 2013 through December 2018	Not published yet (Ridker et al., 2011)
CIRT							

*ACS, acute coronary syndrome; CAD, coronary artery disease; CETP, cholesterol ester transfer protein; LVEF, left ventricular ejection fraction; MI, myocardial infarction. Red boxes indicate trials with negative results, green boxes those with (at least partially) positive results, whithe boxes, indicate inconclusive results, and gray boxes indicate trials under way*.

## Modulation of inducers

**Figure 1:**
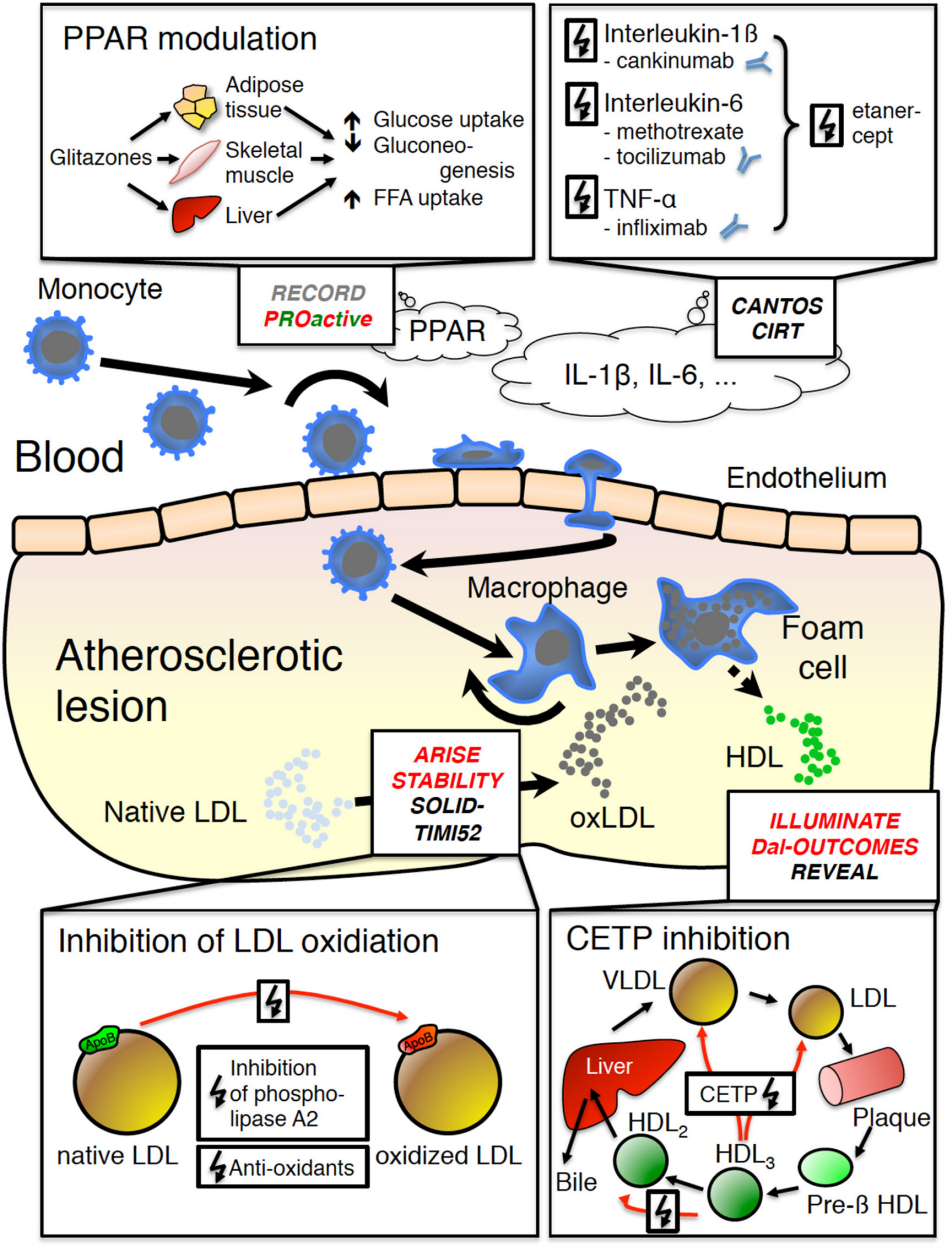
**Basic mechanisms of atherogenesis and studies specifically targeting inflammation in atherogenesis**. Briefly, blood monocytes enter the subendothelial space where they differentiate toward macrophages and foam cells, which in turn secrete pro-inflammatory mediators leading to further inflammation, plaque growth and eventually plaque rupture. RECORD (rosiglitazone) and PROActive (pioglitazone) have studied the role of peroxysome proliferator-activated receptor-γ (PPARγ) agonists, which increase insulin sensitivity, glucose and free fatty acid (FFA) uptake, while reducing gluconeogenesis including anti-inflammatory effects. CANTOS (canakinumab) and CIRT (low dose methotrexate) target IL-1β or IL-6. ARISE (succinobucol), STABILITY and SOLID-TIMI52 (both darapladib) target oxidation of native LDL, which significantly contributes to foam cell formation. ILLUMINATE (torcetrapib), Dal-OUTCOMES (dalcetrapib) and REVEAL (anacetrapib) target HDL levels via inhibition of the cholesterol ester transfer protein (CETP), which is potentially atheroprotective. Red font: negative trial results; red-green font: partially positive results, substance abandoned for side effects; black font: results pending. CETP cholesterol transfer protein; FFA, free fatty acids; HDL, high density lipoprotein; IL, interleukin; LDL, low density lipoprotein; oxLDL, oxidized low density lipoprotein; PPAR, peroxysome proliferator-activated receptor; VLDL, very low density lipoprotein.

Agents that modulate inducers of atherosclerosis largely focus on lipid metabolism (Figure 1). In fact, antioxidants, inhibitors of lipoprotein-associated phospholipase A_2_ (Lp-PLA_2_), niacin, and cholesterol ester transfer protein (CETP) inhibitors either aim at decreasing LDL levels or increasing HDL levels or both. This reflects the important role of lipids during atherogenesis. Thus, LDL and modified forms of LDL are able to promote leukocyte recruitment to the arterial wall (Gleissner et al., 2007). Similarly, both native and oxidized LDL induce macrophage foam cell formation leading to secretion of various pro-inflammatory cytokines and chemokines that in turn promote plaque development and destabilization (Shashkin et al., 2005; Cho et al., 2007).

### Antioxidants

Oxidative modification of LDL has been shown to be an essential mechanism increasing their inflammatory potential (Steinberg, 2009). Accordingly, the use of anti-oxidants may be a suitable approach to prevent adverse cardiovascular events. Despite promising results of *in vitro* experiments with vitamin E (a potent antioxidant), clinical trials have failed to achieve positive results in coronary artery disease patients (Wallert et al., 2014). Succinobucol is a probucol derivative with anti-inflammatory and antioxidant properties (Muldrew and Franks, 2009). The Aggressive Reduction of Inflammation Stops Events (ARISE) study investigated whether succinobucol reduced adverse cardiovascular events in 6000 patients early after acute coronary syndrome. Unfortunately, succinobucol had no significant effect on the primary endpoint (Tardif et al., 2008).

### Inhibition of lipoprotein-associated phospholipase A_2_ (LP-PLA_2_)

Modification of LDL particles can be mediated by phsopholipase A_2_ (PLA_2_). Phospholipases may promote inflammation by producing precursors of arachidonic acid from membrane glycerophospholipids (Corson, 2009). Briefly, LP-PLA_2_ may hydrolyse oxidized phospholipids resulting in pro-inflammatory mediators that lead to endothial dysfunction and plaque progression (Lp-PLA_2_ Studies Collaboration et al., 2010). There is a soluble form (sPLA_2_) and a lipoprotein-associated form (Lp-PLA_2_). A large meta-analysis including 79,036 patients demonstrated that the Lp-PLA_2_ mass and activity are correlated with the risk for cardiovascular disease (Lp-PLA_2_ Studies Collaboration et al., 2010). Darapladib inhibits Lp-PLA_2_ activity by roughly 95%, has no effects on blood lipids and has been shown to lower hs-CRP and IL-6 levels (Mohler et al., 2008). Furthermore, both in a porcine model of atherosclerosis and in human individuals, darapladib significantly reduces the necrotic core of atherosclerotic plaques (Serruys et al., 2008). Accordingly, PLA_2_ inhibition by dalcetrapib has been suggested to be a suitable approach to prevent cardiovascular events.

Thus, in the STabilization Of Atherosclerotic Plaque By Initiation of DarapLadIb TherapY (STABILITY) trial darapladib was tested in 15,828 patients with stable CAD and one risk factor for its ability to reduce a composite endpoint consisting of cardiovascular death, non-fatal myocardial infarction, or stroke. Similarly, the Stabilization Of pLaques usIng Darapladib—Thrombolysis in Myocardial Infarction 52 (SOLID-TIMI52) trial tested the effects of darapladib on adverse cardiovascular events early (≤30 days) after acute coronary sondrome (13,000 patients) (White et al., 2014). While the STABILITY failed to show a significant effect of darapladib on adverse cardiovascular events (White et al., 2010), results from the SOLID-TIMI52 trial are still pending.

### Niacin

Niacin, a member of the vitamin B family, is known to lower LDL and increase HDL levels (Jackevicius et al., 2013). Despite clear evidence, niacin has been increasingly used in coronary artery disease patients. A large randomized controlled clinical trial has now investigated its role as an anti-atherosclerotic drug: The effects of niacin were studied in the Heart Protection Study 2–Treatment of HDL to Reduce the Incidence of Vascular Events (HPS2-THRIVE) study. In this study, 25,673 high risk CAD patients were treated with standard lipid-lowering therapy plus placebo or plus extended-release niacin with laropiprant for 3.9 years (Group et al., 2014). Laropiprant was added to reduce flush symptoms known to occur after niacin intake. Other than expected, niacin did not affect the composite end point of major cardiovascular events, but increased the incidence of diabetes and other side effects such as gastrointestinal, musculskeletal, or skin problems, and infections.

### Cholesterol ester transfer protein (CETP) inhibition

There is clear epidemiological evidence that increased high density lipoproteins (HDL) plasma levels are associated with beneficial outcome in coronary artery disease (Assmann et al., 2002). By contrast, low HDL levels are clearly correlated with adverse cardiovascular outcome making HDL an excellent biomarker for risk prediction (Toth et al., 2013). Therefore, HDL levels have come into focus as a potential therapeutic target. It is thought that HDL acts in an anti-inflammatory manner via different pathways: Most importantly, HDL is able to mediate cholesterol efflux from macrophage foam cells via the cholesterol efflux transporters ABCA1 and ABCG1 thereby modifying the inflammatory response (Landmesser et al., 2012). Furthermore, HDL may have direct anti-inflammatory effects on endothelial cells (Wu et al., 2013) and macrophages (De Nardo et al., 2014). Accordingly, raising HDL levels has been proposed to be beneficial and potentially prevent adverse cardiovascular events.

A series of studies has tested inhibitors of the CETP, which increase HDL levels. CETP mediates the transfer of cholesteryl esters from HDL to VLDL and LDL thereby decreasing HDL levels. CETP inhibition in turn increases HDL levels. Human CETP could be shown to increase atherosclerosis in mice (de Vries-van der Weij et al., 2009) Furthermore, there are data suggesting that the CETP inhibitor anacetrapib may reduce atherosclerosis and promote plaque stability in mice (Kuhnast et al., 2014).

Two substances—torcetrapib and dalcetrapib—have been invstigate in two large clinical trials [ILLUMINATE (15,067 patients), Dal-OUTCOMES (15,871 patients)]. In fact, both significantly increased HDL levels, however, in the case of torcetrapib, excess mortality was seen—most likely due to off-target effects such as increased blood pressure (Barter et al., 2007). By contrast, dalcetrapib significantly increased HDL levels without modulating other cardiovascular risk factors overall resulting in no significant reduction of adverse cardiovascular events (Schwartz et al., 2012). The effects of anacetrapib on the course of CAD are currently under investigation (REVEAL, 30,000 patients)—results are expected in 2017 (Landmesser et al., 2012).

One explanation why thus far CETP inhibition has failed to prevent cardiovascular events may be given by a recently published Mendelian randomization study, which showed that genes increasing HDL levels do not decrease cardiovascular risk (Holmes et al., 2014). Furthermore, there are increasing data suggesting that HDL functionality may significantly differ between healthy individuals and CAD patients—in fact, HDL isolated from CAD patients may even increase inflammation via eNOS inhibition and loss of its anti-inflammatory potential (Besler et al., 2011). Thus, merely increasing HDL may not be sufficient to reduce cardiovascular risk. It remains to be seen whether the approach of CETP inhibition is feasible after all or whether the increase of HDL achieved by CETP inhibitors will generally fail to prevent adverse cardiovascular events. If that were the case, other options to affect HDL may be suitable targets. However, the focus may shift from merely increasing HDL levels to specifically improving HDL functionality. At this point neither appropriate tools to measure HDL functionality in clinical practice, nor specific agents to affect HDL functionality are readily available.

## Modulation of effectors

Compared to therapies that modulate inducers of atherosclerosis, current therapeutic approaches that target modulation of effectors are scarce. It is surprising to see that none of the approaches target receptors such as CD36 or SR-A. CD36 and SR-A account for the vast majority of modified LDL uptake (Kunjathoor et al., 2002). SR-A is a receptor for acetylated LDL, whereas CD36 binds both acetylated LDL and oxidized LDL (Kunjathoor et al., 2002). Deficiency of CD36 or SRA-A or both have been associated with reduced atherogenesis in *Apoe*^−/−^ mice (Babaev et al., 2000; Febbraio et al., 2000), however, there was no additive effect on lesion development when both were knocked out (Kuchibhotla et al., 2008). In some of these studies, scavenger receptor deficiency was associated with reduction of foam cell formation or lesion complexity without affecting lesion size (Moore et al., 2005; Manning-Tobin et al., 2008). These different findings may be explained by differences in genetic backgrounds, time points assessed, and different methods used to measure atherosclerosis (Witztum, 2005). Furthermore, one has to bear in mind that receptors such as CD36 are ubiquitously expressed and relevant to many disease processes—e.g., CD36 not only is a major receptor for oxidized LDL, but also plays an important role in malaria pathogenesis. Taken together it seems unlikely that these targets are specific enough to achieve satisfactory results.

Similarly, it is surprising that there are no large randomized controlled trials investigating the effects of anti-rheumatic drugs on atherosclerosis. For example, a retrospective cohort of 2101 rheumatoid arthritis patients showed that treatment with methotrexate was associated with a significantly reduced incidence of a combined endpoint including myocardial infarction, unstable angina or coronary revascularization (Bili et al., 2014). At this point, data on antibodies such as infliximab, etanercept, or tocilizumab seem inconclusive: Most studies are rather small, they investigate “soft” end points such as arterial stiffness (Tam et al., 2012) or look at pre-specified patient groups that only partially reflect “real world” patients in cardiovascular medicine (Nieuwdorp et al., 2009; Yeh et al., 2014), some studies are negative (Ramonda et al., 2014).

### Insulin sensitizers

Insulin sensitizers are a group of drugs activating the peroxisome proliferator-activated receptor-γ (PPARγ). It has been known for a long time that the transcription factor PPARγ is involved in both metabolic processes and vascular inflammation (Neve et al., 2000). Through activation of a number of metabolic genes, PPARγ agonists increase insulin sensitivity especially in liver, muscle and fat tissue thereby increasing glucose uptake from the blood. Based on pathophysiological considerations, it was postulated that PPARγ not only decreases blood glucose but in addition may decrease the risk of cardiovascular events. There are two large randomized controlled clinical trials testing the effects of rosiglitazone and pioglitazone on cardiovascular outcome.

In the RECORD trial, 4447 patients with type 2 diabetes on anti-diabetic therapy were additionally treated with rosiglitazone (Home et al., 2009). During a mean 5.5-year follow-up, rosiglitazone turned out to be non-inferior in regards to cardiovascular death, myocardial infarction, or stroke (Neve et al., 2000). This did not confirm a previously published meta-analysis that had claimed an increase of myocardial infarction with rosiglitazone (Nissen and Wolski, 2007).

In the PROactive study 5238 patients with type 2 diabetes and macrovascular disease were either treated with pioglitazone or placebo in addition to standard therapy and followed up for 2.85 years (Dormandy et al., 2005). Pioglitazone significantly reduced the occurrence of fatal or non-fatal myocardial infarction with a moderate *p*-value of 0.045. All-cause mortality and cardiac mortality were not affected by treatment with pioglitazone. Even though, these results were encouraging, pioglitazone should be used with extreme caution as development of bladder cancer has been recognized as serious side effect.

Taken together, even though being an attractive pharmaceutical principle for treating diabetes, PPARγ activation has not been successful in clinical practice—mostly due to safety concerns.

### Inhibition of IL-1β

IL-1β may represent a suitable target to treat atherosclerosis (Dinarello, 2009). Currently, the Canakinumab Anti-inflammatory Thrombosis Outcome Study (CANTOS) investigates the effects of canakinumab, a monoclonal antibody against IL-1β on cardiovascular events in 17,200 patients with prior myocardial infarction. Canakinumab has initially been developed to treat rheumatoid arthritis (Dhimolea, 2010). It has no effects on plasma lipids, but significantly lowers markers of inflammation such as IL-6 or CRP (Ridker et al., 2012). Results of the CANTOS trial are expected in 2016 (Ridker et al., 2011).

### Inhibition of the IL-6 pathway

Interleukin-6 clearly plays an important role in atherogenesis as shown by a meta-analysis of 82 studies published in 2012 (IL6R Genetics Consortium Emerging Risk Factors Collaboration et al., 2012). Low dose methotrexate (which partially acts on the IL-6 pathway as demonstrated by reduction of IL-6 plasma levels) has been shown to reduce cardiovascular events in patients with rheumatoid arthritis with hazard ratios between 0.3 and 0.85 (Ridker, 2009; Micha et al., 2011). Accordingly, in the Cardiovascular Inflammation Reduction Trial (CIRT), 7000 are being treated with 15–20 mg methotrexate per week (including 1 mg of folate) or placebo (plus folate) (Everett et al., 2013). The study will include patients with stable CAD and test the occurrence of a composite endpoint consisting of cardiovascular death, non-fatal MI or stroke. Results can be expected in 2018/19.

## Why have so many studies failed?

In fact, with exception of the large statin trials such as the JUPITER study (Ridker et al., 2008), none of the approaches to specifically treat inflammation in order to prevent or reduce atherosclerosis in human individuals was successful. The fact that statins have pleiotropic effects and act in an anti-inflammatory fashion beyond lowering cholesterol can be considered a lucky coincidence rather than due to intention (Mihos et al., 2014). Based on the considerations made above, one has to wonder why anti-inflammatory agents have widely failed in clinical practice.

The first explanation is pharmacology: Off-target effects may predominate potential anti-atherosclerotic effects as seen with torcetrapib (Barter et al., 2007) or pioglitazone (Ferwana et al., 2013). Furthermore, the chosen targets may be inappropriate—e.g., they may lack specificity. In fact, it is hard to believe that inhibition of a single specific pro-inflammatory cytokine may result in reduced atherosclerosis without serious side effects such as an increase of infections or neoplastic disease. On the other hand, the pathophysiological role of the proposed targets may simply be not be fully understood: e.g., dalcetrapib did significantly increase HDL levels, however, this did not result in a significant reduction of adverse cardiovascular events (Schwartz et al., 2012). Thus, HDL levels alone do not seem to be a useful surrogate marker for prediction of adverse events and one rather needs to test HDL functionality to really make use of it as a therapeutic target (Luscher et al., 2014). Finally, a one-target strategy may simply be insufficient to successfully treat a disease as complex as CAD as there are redundant pathways.

## Conclusions

In conclusion, the aim to prevent or treat atherosclerosis via modulating inflammation remains challenging. The efforts that have been made so far show us two things: Firstly, the application of data obtained from small animal models of atherosclerosis is not necessarily pertinent to the human disease. Secondly, surrogate markers are not necessarily good therapeutic targets to prevent adverse clinical events. Accordingly, careful translation of results from animal studies into the human system and large clinical trials will be needed to successfully improve anti-inflammatory therapy of atherosclerosis in the future. To achieve this ambitious goal two major points need to be considered: (1) Developing animal models that properly reflect the human disease is crucial. This specifically includes models that allow study of plaque rupture and plaque regression. Rabbit models may be helpful in this context, under specific circumstances large animal models may similarly be suitable. Furthermore, novel molecular imaging techniques may allow better insight into the events leading to plaque rupture (Korosoglou et al., 2014). (2) Translation of animal data into the clinical setting needs to be undertaken with extreme caution. As adverse cardiovascular events are quite rare, large studies with long follow-up periods are needed to investigate the effects of novel drugs on hard cardiovascular end points. In many cases, these trials will have to be supported by governmental funding, as industry sponsors may not be willing to risk failure of large long-term studies. Considering the enormous impact of CAD on our societies, it seems worthwhile pursuing this aim.

### Conflict of interest statement

The authors declare that the research was conducted in the absence of any commercial or financial relationships that could be construed as a potential conflict of interest.
